# New York Pathological Society

**Published:** 1866-06

**Authors:** 


### PROCEEDINGS OF MEDICAL SOCIETIES.

### NEW YORK PATHOLOGICAL SOCIETY.

**Stated Meeting of March 28, 1866—Dr. F. H. Hamilton, Pres't, in the Chair.**

Dr. E. Bradley presented a small fragment of the squamous portion of the left temporal bone, removed from the skull of a rebel soldier who had been wounded in that region by a spent ball. Upon the receipt of the injury he lost the power of speech, and remained in that condition for ten days. The fragment of bone was then removed, and material relief followed. A marked hesitancy of speech remained for some time after the operation; but this disappeared by the time that the wound had healed.

Dr. Rogers remarked that the precise situation of the fracture described would be an interesting point to determine, as bearing upon the question of the precise location of the organ of speech. Dr. Bradley was unable to state the exact location of the wound, having received from the gentleman who observed the case no other particulars than those already given.

Dr. Bradley exhibited a specimen of calcified falx cerebri, from a patient, 50 years of age, who had been subject to epileptic convulsions, and who had died of chronic meningitis. The dura mater was thickly sprinkled with the calcified plates.

In connexion with this case the Doctor mentioned a recent patient of his who died of apoplexy at the N. Y. Hospital. He had fallen in convulsions while walking in the street, and had been conveyed, unconscious, to the hospital, with all the symptoms of a well marked apoplectic seizure. The post-mortem examination disclosed a remarkable development of the crista galli, which invaded the left cerebral lobe. The right ventricle was ruptured and filled with clotted blood; and four ounces of thick blood were found beneath the meninges. The question was suggested whether the apoplexy had not been caused by the presence of this bony growth.

Dr. O. H. Smith exhibited a specimen of ulceration of the duodenum, from a young lady, 27 years of age, who suffered, one year ago, from typhoid fever. She recovered in part from this, went into the country, and was slowly regaining her strength, when, in December last, she caught a cold, which was followed by a severe chill and febrile symptoms. Great pain in the stomach and right hypochondrium, with vomiting, ensued; and the pain and vomiting continued for two weeks. Dr. Smith saw her about the first of January last, when she was brought to the city. The pain still continued, and the patient vomited nearly all the ingesta; the pulse was very rapid, and the general appearance of the case unfavorable. Opium and quinine were exhibited, but without effect; and Dr. Smith thought that the lady was probably affected with cancer of the stomach. Her memory and intellectual powers now failed rapidly; near the first of March she had a convulsion, followed by partial dextral hemiplegia; and she remained in this condition until the 22d of March, when she died.

Autopsy twenty-four hours after death. The stomach was found to be healthy. The peritoneal surface was covered with a lymphy exudation, apparently the sequel of an old peritonitis.

Adhesion was found between the abdominal walls and three or four inches of the duodenum; and on breaking it down, pus oozed into the cavity of the abdomen. In the midst of this adhesion, and about six inches from the stomach, there was ulceration and perforation of the gut. This lesion the Doctor could only account for by supposing the duodenal glands to have been poisoned by the typhoid virus.

Dr. Markoe presented the calvarium of a patient whom he had trephined for epilepsy; remarking that the specimen was interesting, not only in reference to the method of treatment employed, but also in connexion with the cranial necrosis that occurred in the history of the case. The patient was a boy, 17 years of age. At the age of five years he received a wound upon the left forehead by being run over; no fracture, however, occurred, and the wound healed rapidly. But in six months after this accident, the boy became rather suddenly paralyzed on the right side. The hemiplegia was complete. He recovered somewhat, after a few weeks, from this condition, but did not entirely regain his former health; he suffered from headache, when the scar on his forehead invariably reddened; his memory was weakened, and his activity diminished; yet he was able to attend school with his fellows. When 14 years old he had epileptiform convulsions, which recurred at intervals of three to seven days; latterly they took place with some regularity on Sundays; and his intellect was sinking into that state which is so often the concomitant of confirmed epilepsy.

Such was the condition of the patient when Dr. Markoe was requested to see him, about five months ago. The case seemed to be one in which the disease might fairly be supposed to depend upon the injury received in childhood; statistics gave a reasonable promise that relief might be afforded by operative procedure; and it was decided to make the attempt, the boy's parents assenting to any plan that gave prospect of benefit to the sufferer. On the 20th October, 1865, two discs of bone were removed by the trephine, and an intervening bridge of bone was displaced by the *ronguer;* leaving an opening in the skull about three-quarters by two inches. The edges of the opening were carefully smoothed. The immediate result of the operation was satisfactory; and no convulsions occurred for a month. A very slight one happened at the end of that time; and then for two weeks longer he was free from epileptic seizure; the wound healing favorably meanwhile. Pyæmia, however, then began to prevail in the hospital; and this patient suffered with the rest. The pus began to burrow under the scalp, in spite of counter-openings and of pressure; the bone became extensively denuded and necrosed; the wound assumed a bad condition, the patient sank, and finally died about a week ago, having been in a comatose state for nearly a fortnight. During this time a fungus cerebri had sprung up and attained the size of a small orange.

The autopsy showed the hernia cerebri as a pultaceous reddish mass. Below that portion of the hemispheres from which it sprung was a tract of soft and greenish-yellow cerebral substance; but no abscess was found, upon careful search, in this part of the brain. On the right side, however, corresponding in situation with the wound upon the left side, was a deep-seated abscess, which contained two ounces of laudable pus. The calvarium was extensively necrosed; *but nature had made no effort to separate the sequestrum;* a circumstance which Dr. Markoe regarded as very remarkable. He had observed this phenomenon in the case of syphilitic necrosis; but never before when the disease was of traumatic origin.

In answer to a question, Dr. M. stated that the dura mater was not invaded by the trephine; and that the ulceration which afterward took place in the membranes, allowing the development of the fungus, was precisely defined by the margins of the opening in the bone.

Dr. Hamilton thought that non-separation, in traumatic necrosis, was the result of extreme enfeeblement of the constitution; and referred to the frequent cases of osteo-myelitis which have been observed during the late rebellion. In these cases, which always occurred in broken or feeble constitutions, the disease frequently progressed so far as to involve the entire bone of an extremity.

Dr. W. Beach, jr., presented a large tumor which had been removed from the abdominal cavity, supposing it to be ovarian, by Dr. Huckenberg, of Coxsackie, N. Y. The patient died thirty-nine hours after the operation.

Dr. Krackowizer, on examining the specimen, remarked that it was a fibrous tumor of the uterus, and thought that a detailed history of the case would be interesting. Dr. Markoe observed that the evident mistake in diagnosis that had been made showed the necessity of a careful examination with the uterine sound previous to operating, in cases of suspected ovarian disease.

Dr. Krackowizer presented a segment of a fore-arm which had been removed by amputation from a soldier who had been wounded at Gettysburg, and whose right arm had been amputated several days after the injury. The stump took several months in healing. When the cicatrization was nearly completed, the patient noticed two nodules upon the stump, small, movable and painful. They increased in size and in sensitiveness, and at last allowed him no rest. By the kindness of Dr. Marsh, Dr. Krackowizer saw the patient at the "Lincoln Home;" concluded that neuroma existed; and as the stump was badly shaped, concluded to remove the disease by reamputation. This being performed, the principal nerves of the extremity were found to terminate in the sensitive nodules already described.

The Society went into executive session.

